# Macrocolonies (Granules) Formation as a Cause of False-Negative Results in the MGIT 960 System: Cause Analysis and Correlation with Mycobacterial Species

**DOI:** 10.1155/2015/501847

**Published:** 2015-11-04

**Authors:** Xia Yu, Liping Zhao, Guanglu Jiang, Yifeng Ma, Weimin Li, Hairong Huang

**Affiliations:** National Clinical Laboratory on Tuberculosis, Beijing Key laboratory on Drug-Resistant Tuberculosis Research, Beijing Chest Hospital, Capital Medical University, Beijing Tuberculosis and Thoracic Tumor Institute, Beijing 101149, China

## Abstract

*Background*. The viable mycobacterial bacilli can sometimes form granules in the Mycobacterium Growth Indicator Tube (MGIT) to produce instrument-negative outcomes when BACTEC MGIT 960 culture is performed. The cause of this phenomenon has never been analyzed. *Methods*. Thirty-one instrument-negative, granule presenting MGIT vials were collected for conducting acid-fast staining and also liquid and solid subculture. Species identification and drug susceptibility test were performed with the recovered strains. Cultivation test was done by inoculating small amount of bacilli into the MGIT vials. *Results*. Twenty-four and twenty-nine of the tested MGIT vials were smear and culture positive, respectively. In total, 18, 4, and 7 of the cultivated strains were identified as *Mycobacterium tuberculosis* complex, *M. intracellulare*, and *M. xenopi*, respectively. When a limited amount of bacilli was inoculated, the granule formation was observed for *M. xenopi* strains in the MGIT system. *Conclusions*. The granules observed in the instrument-negative MGIT vials consisted of viable bacilli, which emphasized the need of visual inspection to increase recovery rate. Limited bacterial load and specific species might be the cause of granule forming.

## 1. Introduction

Bacterial culturing is substantially more sensitive than smear microscopy to diagnose tuberculosis (TB) [1], while liquid culture is faster and more sensitive than solid culture [2–5]. The BACTEC MGIT 960 system (Becton, Dickinson and Company, Franklin Lakes, NJ, USA) is a widely used commercial automatic culture system based on the bacterial culturing in broth. A situation may occasionally arise when using MGIT 960 system, that is, the presence of visible granules (characterized by a scanty number of round yellow-pigmented appearance and approximately 1 mm size), in some instrument-negative vials. Even after shaking the MGIT tubes, these granules maintain their shapes rather than forming a homogenously turbid solution. To better understand this outcome, we collected such kind of vials to analyze the features and cause of these granules.

## 2. Material and Methods

### 2.1. Sample Collection

Mycobacterial culturing by MGIT 960 system was performed for the 2,410 clinical specimens, collected during January 2010 to December 2010 at the National Tuberculosis Clinical Laboratory of Beijing Chest Hospital. From these samples, 1,338 were flagged as culture positive by the instrument.

### 2.2. Smear Microscopy Examination

Granules in the MGIT vials were harvested by centrifugation and swabbed directly with sterile Pasteur pipettes onto slides to prepare smears. The slides were then stained using the Ziehl-Neelsen method, according to the protocol by World Health Organization (WHO) [6].

### 2.3. Subculture

For each of the tested MGIT vials, the tube was briefly centrifuged and the sediment was resuspended in 1 mL of 7H9 broth. Half of the suspension was then inoculated into a fresh MGIT vial while another half was inoculated onto a fresh LJ medium slant for mycobacterial culture.

### 2.4. Species Identification

The 16S-23S rRNA internal transcribed spacer (ITS) regions of the strains were sequenced after amplification by PCR to categorize the strains into species, as mentioned elsewhere [7–9].

### 2.5. Drug Susceptibility Test (DST)

The DST was performed by the MGIT 960 system as recommended [10, 11]. The tested drugs included isoniazid (INH), rifampin (RIF), streptomycin (SM), and ethambutol (EMB).

### 2.6. Seeded Culture Studies

*Mycobacterium tuberculosis* strain H37Rv (ATCC strain 27294) and 8 aforementioned isolates acquired from the granules' subculture were chosen by the method of stratified cluster sampling [12]. The 8 strains included 2* M. tuberculosis* complex (*MTC*) strains, 3* M. xenopi* strains, and 3* M. intracellulare* strains. The isolates were grown on LJ slants at 37°C for three weeks; then, the bacterial suspensions were prepared using 0.5% (vol/vol) Tween 80 in 0.9% sodium chloride with turbidity equal to the McFarland number 1 turbidity standard. Next, the suspensions were diluted to 10^−5^, 10^−6^, 10^−7^, and 10^−8^ of the McFarland number 1 turbidity standards. Aliquots (approximately 100 *μ*L) of each preparation were inoculated into new MGIT vials in duplicate. The MGIT 960 culture was considered positive if the instrument signaled positive and the following microscopic examination proved AFB existence. If the instrument did not signal positive until after 6 weeks of incubation, the tubes were visually inspected for the presence of granules. All the inoculated MGIT vials were centrifuged after the culture completion and sediments were used for acid-fast staining. The existence of viable mycobacteria was proven for all the instrument-negative vials (including the granule forming vials) by inoculating the sediments into new MGIT960 vials for another culture.

### 2.7. Statistics

Chi-square test was performed for comparing the species composition between instrument positive vials and granule forming vials. A value of *P* < 0.05 was considered statistically significant. The statistical analysis was carried out using SPSS version 13.0.

### 2.8. Ethical Considerations

The study was approved by the ethical committee of Beijing Chest Hospital. The data was accessible only to study personnel.

## 3. Results

### 3.1. Microscopy Examination and Culture

**Figure 1:**
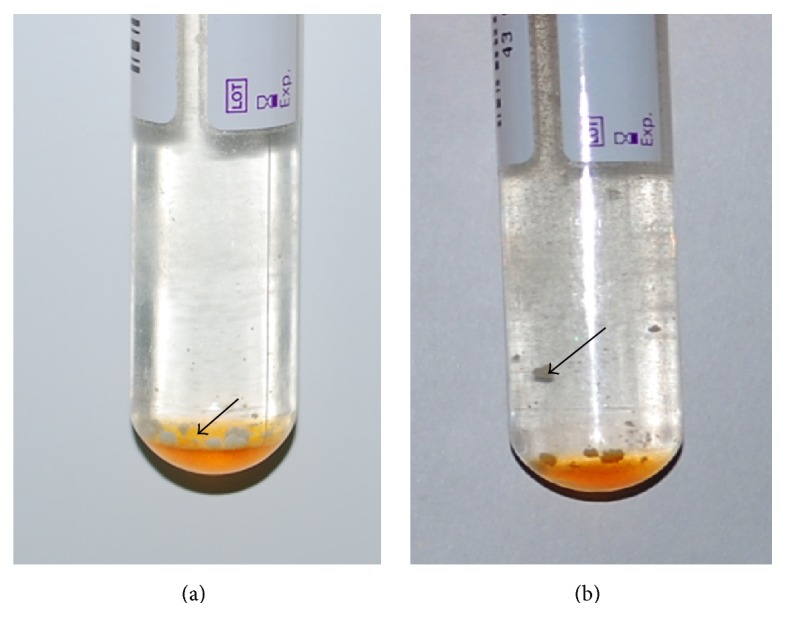
Granules in the instrument-negative MGIT vials. (a) Granules before shaking. (b) Granules after shaking.

A total of 31 instrument-negative MGIT vials, with visible granules, were collected (Figure 1). Among these, the GI values for 27 vials were zero and were 40, 5, 63, and 55 for the other 4 vials. All the granules vials remained instrument-negative even after elongating the incubation time for 2 more weeks in the MGIT960 system. The following microscopic examination, MGIT 960 subculture and LJ medium subculture produced positive outcomes for 24, 25, and 29 vials, respectively.

### 3.2. Species Identification Outcomes

Among the 1338 instrument flagged-positive MGIT vials, the species composition was as follows:* MTC* (*n* = 1267),* M. intracellulare* (*n* = 31),* M. abscessus* (*n* = 17),* M. kansasii *(*n* = 11),* M. gordonae* (*n* = 6),* M. avium* (*n* = 3),* M. xenopi* (*n* = 1),* M. malmoense* (*n* = 1), and* M. scrofulaceum* (*n* = 1). Among the strains recovered from granules,* MTC*,* M. intracellulare*, and* M. xenopi* accounted for 62.07% (18/29, *χ* ^2^ = 53.58, *P* < 0.001), 13.79% (4/29, *χ* ^2^ = 14.99, *P* < 0.001), and 24.14% (7/29, *χ* ^2^ = 282.50, *P* < 0.001) of the strains, respectively.

### 3.3. Drug Susceptibility Test Outcomes

Among the 18 aforementioned* MTC* strains, 7 strains were sensitive to all the four tested drugs (INH, RIF, SM, and EMB) and 7 strains were classified as MDR strains whereas the remaining 4 strains demonstrated resistance patterns other than MDR. The drug susceptibility test outcomes of 18* MTC* isolates were listed in Table 1.

### 3.4. Inoculation Test

The outcomes of inoculation test for 3* M. xenopi*, 3* M. intracellular*, and 2* MTC* strains as well as for* M. tuberculosis* H37Rv are listed in Table 2. The vials signaled as culture negative, with or without granule formation, were all culture positive after subculturing the sediments into the new MGIT vials.

## 4. Discussion

The BACTEC MGIT 960 instrument is a fully automated, noninvasive system that is suitable for the detection of tuberculosis and other mycobacterial species. We observed that some instrument-negative tubes presented visible granules after 6 weeks of incubation; however, extending the incubation time to 8 weeks did not produce any positive outcome. Bacteriological examination proved that the granules consisted of viable bacilli. Meanwhile, other studies have also reported failure in growth detection by MGIT 960 [13–15]. Although the manufacturer suggested either subculturing the vials with granules or conducting acid-fast stains and treating the specimen as presumptive positives (provided that either test is positive) [16]. The reason for granule formation is not well understood so far: dead bacilli cannot be the cause because 29 of the 31 subcultured vials with granules achieved positive culture outcomes. Therefore, according to our work, visual inspection of the negative vials could further increase approximately 1% of bacilli recovery rate for MGIT 960 culture.

Our DST outcomes suggested that the granule formation may not be related with drug resistance patterns, since the MDR-TB proportion is similar to that of the overall isolates in the same laboratory (approximately 1/3 of the test strains are MDR strains). Species identification demonstrated that 11 strains (11/29, 37.93%) were NTM strains, which suggested that the NTM is not the exclusive explanation for granule formation. However, our data did show that some types of NTM might have a better opportunity to form granules than others, because* M. intracellulare* or* M. xenopi* accounted for much higher percentages of granule forming isolates than the general culture positive isolates (*P* < 0.001). On the other hand,* MTC* isolates demonstrated opposite tendency for granule formation (*P* < 0.001). A similar study demonstrated that 94% (129/137) of instrument-negative outcome was caused by NTM, and the most frequent NTM was* M. avium complex* and* M. gordonae* in the United States [14]. However, due to limited total number of tested strains in our study, the possibility that this result was due to sampling error cannot be confidently excluded.

In the inoculation assay, only the 3* M. xenopi* strains produced granules when inoculated with limited bacterial loads. Such outcomes not only proved that granule formation might be a specific growing characteristic for* M. xenopi* but also demonstrated that low bacterial load might be a synergetic factor. Piersimoni et al., also found that due to granular pattern of growth (which keeps oxygen consumption below the detection threshold), MGIT 960 can fail in the detection of* M. xenopi* bacilli [13]. The* MTC* strains and* M. intracellulare* strains failed to produce granules with low inoculation bacterial loads, demonstrating that some other factors may also exist. Among the related patients with granule forming outcomes, except for one with unclear medical record, all other patients were receiving anti-TB treatment when the samples were collected. The treatment duration lasted from 1 month to more than 2 years. Therefore, it is likely that the administration of anti-TB therapy (probably together with a low bacterial load) can be a key factor for the development of granules. Among the granule forming vials, 3 with* MTC* granules and 2 with* M. xenopi* granules originated from same patients within a very short time period. We guess the bacterial loads in specimens might have been similar during the short period.

Our experiment also demonstrated that acid-fast staining increased the chances to identify the AFB in the granules, as 24 (24/29, 82.76%) of the granule forming vials were smear positive. Since it is possible to lose AFB staining while preparing smear slides with liquid broth, improving the smear preparation process can also increase the AFB detection sensitivity.

## 5. Conclusions

The granules observed in the instrument-negative MGIT vials consist of viable bacilli; therefore, visual inspection for granules is suggested for better yield. Limited bacterial load and specific species can also cause granule formation during MGIT 960 culturing.

## Figures and Tables

**Table 1:** The drug susceptibility test outcomes of 18 *MTC* isolates with granules forming to four first-line antituberculosis drugs.

Antituberculosis drugs				Number of isolates
SM	INH	RIF	EMB	
S	S	S	S	7
R	R	R	S	5
R	S	S	S	2
R	R	R	R	2
R	R	S	S	1
S	R	S	S	1

S: susceptible; R: resistant.

**Table 2:** The MGIT culture outcomes when inoculated with different bacterial loads in duplicate (by tubes).

Number and species	Diluted McFarland turbidity standard	Culture positive	Turn-around time (days) (means ± SD)	Culture negative with granules forming	Culture negative without granules forming
3 *M*. *xenopi *strains	10^−5^	6/6	9.67 ± 1.03	0/6	0/6
	10^−6^	2/6	13.5 ± 0.71	4/6	0/6
	10^−7^	1/6	26.3 ± 0.57	5/6	0/6
	10^−8^	0/6	—	6/6	0/6

3 *M*. *intracellulare* strains	10^−5^	6/6	9.17 ± 1.47	0/6	0/6
	10^−6^	4/6	11.75 ± 1.26	0/6	2/6
	10^−7^	0/6	—	0/6	6/6
	10^−8^	0/6	—	0/6	6/6

2 *MTC* strains	10^−5^	4/4	11.00 ± 0.82	0/4	0/4
	10^−6^	4/4	15.00 ± 0.81	0/4	0/4
	10^−7^	3/4	25.00 ± 1.00	0/4	1/4
	10^−8^	1/4	36.00	0/4	3/4

H37Rv	10^−5^	2/2	10.00 ± 0.00	0/2	0/2
	10^−6^	2/2	13.50 ± 0.71	0/2	0/2
	10^−7^	2/2	25.00 ± 1.41	0/2	0/2
	10^−8^	0/2	—	0/2	2/2

SD: standard deviations.
